# Macrophage-Derived Exosomes Promote Bone Mesenchymal Stem Cells Towards Osteoblastic Fate Through microRNA-21a-5p

**DOI:** 10.3389/fbioe.2021.801432

**Published:** 2022-01-05

**Authors:** Kun Liu, Xin Luo, Zhao-Yong Lv, Yu-Jue Zhang, Zhen Meng, Jun Li, Chun-Xiu Meng, Hui-Fen Qiang, Cai-Yao Hou, Lei Hou, Feng-Zhen Liu, Bin Zhang

**Affiliations:** ^1^ Department of Oral and Maxillofacial Surgery, School and Hospital of Stomatology, Shandong University and Shandong Provincial Key Laboratory of Oral Tissue Regeneration and Shandong Engineering Laboratory for Dental Materials and Oral Tissue Regeneration, Jinan, China; ^2^ Liaocheng People’s Hospital, Medical College of Liaocheng University, Liaocheng, China; ^3^ Department of Materials Science and Engineering, Liaocheng University, Liaocheng, China

**Keywords:** macrophage, exosomes, microRNA-21a-5p, BMSCs, osteogenic differentiation

## Abstract

The effective healing of a bone defect is dependent on the careful coordination of inflammatory and bone-forming cells. In the current work, pro-inflammatory M1 and anti-inflammatory M2 macrophages were co-cultured with primary murine bone mesenchymal stem cells (BMSCs), *in vitro*, to establish the cross-talk among polarized macrophages and BMSCs, and as well as their effects on osteogenesis. Meanwhile, macrophages influence the osteogenesis of BMSCs through paracrine forms such as exosomes. We focused on whether exosomes of macrophages promote osteogenic differentiation. The results indicated that M1 and M2 polarized macrophage exosomes all can promote osteogenesis of BMSCs. Especially, M1 macrophage-derived exosomes promote osteogenesis of BMSCs through microRNA-21a-5p at the early stage of inflammation. This research helps to develop an understanding of the intricate interactions among BMSCs and macrophages, which can help to improve the process of bone healing as well as additional regenerative processes by local sustained release of exosomes.

## Introduction

Rehabilitation of extensive bone defects is a formidable challenge in modern medical science. However, owing to their outstanding capacity for bringing about osteogenic differentiation, bone marrow mesenchymal stem cells (BMSCs) have become the osteogenous seed cells of bone tissue engineering and cytotherapy in recent years (Li Z. et al., 2020; Sui et al., 2019). The regeneration activities of BMSCs may be attributed to the diverse mechanisms of molecular cross-talk between BMSCs and adjacent cells (Thurairajah et al., 2017). Recent studies have determined that the macrophage-BMSCs crosstalk is substantially useful for the healing of bone defects (Pajarinen et al., 2019; Vallés et al., 2020).

Macrophages are an important part of innate immunity. Depending on the environment macrophages display different phenotypes. The two major phenotype classifications include M1 (pro-inflammatory, classically activated macrophages) and M2 (anti-inflammatory or alternative activated macrophages). This functional plasticity of macrophages has been conceptualized as macrophage polarization. Furthermore, the polarization state of macrophages is fluid and can rapidly sense changes in the microenvironment and switch between M1 and M2 (Muñoz et al., 2020; Li J. et al., 2020). In an *in vitro* setting, exposure to Th1 cytokines, like TNF-α and IFN-γ or lipopolysaccharide (LPS), and polarizes macrophages into the M1 phenotype. Th2 cytokines, for instance, IL-4, and IL-13 polarize the M2 phenotype. The overall balance of M1/M2 macrophages controls the ultimate fate of organs in inflammation or injury. M1 macrophages increase the release of pro-inflammatory cytokines when an organ is infected or inflamed. In the later stages of inflammation, M2 macrophages secrete anti-inflammatory cytokines to suppress the inflammation and promote tissue repair (Selders et al., 2017; Krzyszczyk et al., 2018; Oishi and Manabe, 2018; Shapouri-Moghaddam et al., 2018; Meng et al., 2021). The imbalance of the inflammatory milieu, therefore, leads to abnormal bone repair during bone healing (Vallés et al., 2020). A recent study found that co-cultured macrophages and mesenchymal stem cells can be induced to undergo osteogenic differentiation, with M2 macrophages being the most successful at promoting the generation of new bone tissue (Loi et al., 2016; Zhang et al., 2017; Nathan et al., 2019). Furthermore, quite a few studies have shown that an initial proinflammatory phase modulated by M1 macrophages promotes osteogenesis in BMSCs (Tu et al., 2015; Lu et al., 2017; Romero-López et al., 2020; Xia et al., 2020). However, the mechanism of M1 macrophages promoting osteogenic differentiation at the early stage of inflammation is still rather ambiguous.

Exosomes, as key components of the macrophage-derived cell-conditioned medium, may play an important role. Exosomes are membrane microvesicles with a diameter of 30–200 nm that are derived from endosomes. Exosomes, which carry microRNAs (miRNAs), can be released into their environment (media) and then taken up by distal cells, in which they regulate biological processes (Li et al., 2017; Li and Jiang, 2019). Xiong et al. discovered that there was a substantial overexpression of microRNA-5106 in exosomes derived from M2 macrophages, but not in those derived from M1 macrophages, and that BMSCs osteogenic differentiation would be promoted by the exosomal miRNA by direct targeting of the Salt-inducible kinase 2 and 3 (SIK2 and SIK3) genes (Xiong et al., 2020). However, Xie et al. found that M1 macrophage exosomes support cell osteogenic differentiation (Xia et al., 2020).

This study aimed to see how macrophage-derived exosomal miRNAs affected BMSCs differentiation during the early stages of the pro-inflammatory response. We demonstrated that the miR-21a-5p is highly enriched in exosomes secreted by M1 macrophages and that it can be transferred to BMSCs to induce osteoblastic differentiation *in vitro*. Lian et al. show that miR-21-5p may be a potential pro-osteogenesis regulator (Lian et al., 2018). In addition, Huang et al. found that miR-21-5p targets SKP2 to reduce osteoclastogenesis in a mouse model of osteoporosis (Huang et al., 2021). MiR-21a-5p is highly homologous with miR-21-5p (Budak et al., 2016). To suggest new strategies for promoting bone regeneration, we investigated the molecular mechanism of M1 macrophages promoting the bone formation of BMSCs at an early stage. These findings will enhance our understanding of macrophage-BMSCs crosstalk at the cellular-molecular level and contribute to the development of treatments of bone defects.

## Materials and Methods

### Macrophage Polarization and Preparation of CMs

Murine RAW264.7 (ATCC, United States) was incubated in a complete medium (DMEM; Gibco, Grand Island, United States) supplemented with streptomycin/penicillin (1%, Hyclone, United States) and fetal bovine serum (10%, FBS; Gibco, Grand Island, United States). In each well of a six-well plate, 1 × 10^6^ cells were plated for each group. Macrophages were incubated with 100 ng/ml Lipopolysaccharide (LPS, Solarbio, China) and 20 ng/ml INF-γ (Beyotime, China) to induce M1 polarization, while 20 ng/ml IL-4 (Peprotech, United States) induced M2 polarization (Zhang et al., 2017; Xia et al., 2020). As a control, M0 macrophages were incubated in a complete culture medium. The cells were washed three times in PBS followed by incubation with a complete medium after 24 h. The medium supernatants derived from macrophage were centrifuged after collection for 20 min at 1950 g after another 24 h of culturing. The conditioned medium (CM) comprising M0, M1, and M2 cell cultures were given the respective names CM0, CM1, and CM2. As a control, the entire medium (Norm) was used. Quantitative real-time polymerase chain reaction (qRT-PCR), flow cytometry, and fluorescence staining and imaging were used to identify the phenotypes of polarized cells (as activated by IL-4 or LPS plus INF-γ) and unpolarized cells (complete culture medium).

### Isolation and Identification of BMSCs

Sipeifu Biotechnology Co., Ltd provided C57BL/6J mice (4 weeks old, male) (Beijing, China). The Animal Use and Care Committee of the Liaocheng People’s Hospital inspected and approved the entire animal work. Removal of C57BL/6J mice’s femurs and tibias was carried out aseptically. The bone marrow tissues were flushed and incubated in α-MEM medium supplemented with streptomycin (1%), penicillin (1%), and FBS (10%). The cells cultures were maintained for 7–10 days, with the medium being changed every 3 days. BMSCs were identified by flow cytometry and used in subsequent experiments.

### Isolation and Identification of Macrophage-Derived Exosomes

#### Isolation of Macrophage-Derived Exosomes

RAW264.7 cells were incubated in a polarization medium for 24 h before being washed twice with PBS to eliminate any residual cytokines. After that, the cells were cultured in α-MEM (Gibco, Grand Island, United States) supplemented with 10% exosome-depleted FBS and 1% penicillin/streptomycin. FBS was centrifuged at 100,000 g for 18 h to extract bovine exosomes using a Beckman Optima XE L-80 XP ultracentrifuge (45 Ti rotor) (Beckman Coulter. Inc., United States). The CM formed by the M0, M1, and M2 macrophages was collected separately after 24 h. To extract cells and debris, individual CM specimen was centrifuged at 2,000 g for 30 min at 4°C. The complete exosome isolation (from cell culture medium) reagent was used to separate exosomes from CM samples (Invitrogen, Carlsbad, CA, United States) (Romero-López et al., 2020). Xia et al. used a similar method to extract the exosomes secreted by macrophages (Xia et al., 2020). The CM was moved to a new tube, and each CM supernatant received 0.5 volume of total exosome isolation reagent. Vortex was used to thoroughly combine the mixtures, followed by their incubation at 4°C overnight. After incubation, centrifuge the samples for 1 h at 4°C at 10,000 × g. Finally, 1 ml CM exosomes (designated M0-Exos, M1-Exos, and M2-Exos) were suspended in 100 μl PBS.

#### Identification of Macrophage-Derived Exosomes

The M2-Exos, M1-Exos, and M0-Exos were directed to Novogene Co., Ltd. in Beijing, China, for identification, which included transmission electron microscope (TEM) observation, nanoparticle tracking analysis (NTA), and identification of precise markers by flow cytometry for nanoparticle analysis (NanoFCM).

For one minute, exosomes were dropped on copper. The floating liquid was soaked by the filter paper after 10 μl of uranyl acetate was applied dropwise to the copper mesh for 1 min for precipitation. The grids were dried at room temperature for a few minutes. A Hitachi HT-7700 transmission electron microscope was used to examine the samples (Tokyo, Japan).

Nanoparticle tracking analysis (NTA) was employed for the assessment of particle size and exosome concentration. N30E was used to weigh exosomes (NanoFCM, Xiamen, China). Specific markers CD81 and CD63 were extracted from M1-Exos and M2-Exos to detect the expression of exosome protein.

#### Internalization of the Exosomes by BMSCs

The exosomes were labeled by employing PKH67 fluorescent cell linker kits (Sigma-Aldrich, St. Louis, MI, United States) following the protocol from the manufacturer. In a nutshell, 20 μl of exosomes were diluted in 1 ml of diluent C and 6 μl of PKH67 dye. The labeled exosomes were washed in PBS for 70 min at 100,000 g. Finally, BMSCs were incubated with PKH67-labeled exosomes for 6 h at 37°C. The uptake of exosomes by BMSCs was observed making use of a confocal laser microscope (FV1000; Olympus, Tokyo, and Japan).

#### Effects of Macrophage-Derived Exosomes on BMSCs Osteogenic Differentiation

BMSCs were seeded in 24-well plates, at a density of 2 × 10^5^ cells per well. α-MEM full medium (with 10% FBS and 1% penicillin and streptomycin) was supplemented with 10 nM dexamethasone, 50 μg/ml vitamin C and 10 mM glycerophosphate to make osteogenic medium. To the osteogenic media employed for culturing BMSC, M0-Exos, M1-Exos, or M2-Exos were added at a concentration of 1 μg/ml (Qian et al., 2020). Nothing was added to the osteogenic medium as a blank control (CON). The cells were harvested after 7 days to be stained for alkaline phosphatase (ALP) and qRT-PCR analysis. The cells were harvested after 14 days for alizarin red S staining and activity analysis.

#### Microarray Analysis

The Illumina se50 platform was used to sequence RNA from M1-Exos and M2-Exos (NEB, United States). The Agilent Bioanalyzer 2,100 system’s RNA Nano 6000 Assay Kit (Agilent Technologies, CA, United States) was utilized to test RNA integrity. Multiplex Small RNA Library Prep Set for Illumina (NEB, United States) was employed for building sequencing libraries, and index codes were added to each sample’s sequences. DNA High Sensitivity Chips were used to evaluate library efficiency on the Agilent Bioanalyzer 2,100 device. Small RNAs were reverse transcribed, amplified, and sequenced on the Illumina HiSeq 2,500 platform, which provided 50-bp single-end reads. The samples were analyzed by DESeq based on negative binomial distribution and evaluated by fold change and Qvalue. The differential microRNAs were screened with Qvalue < 0.05 and log2 (foldchange) > 1. The overall distribution of differentially represented miRNAs is inferred using a volcanic map.

#### RAW264.7 Transfection

##### Lentiviral Infection Induces Knockdown and Overexpression of miR-21a-5p

The Gene Corporation provided the lentivirus Lv-mmu-miR-21a-5p-knockdown, overexpression and the negative regulation lentivirus (Genechem Co., Ltd, Shanghai, China). Following incubation for 24 h at 37°C, the RAW264.7 cells were plated into 6-well plates at a density of 2 × 10^5^ cells/well and grown to 20–40% confluence. The RAW264.7 cells were then infected with the lentivirus at a multiplicity of infection (MOI) equivalent to 20. The cells in the shNC,Lv-NC group were transfected with NC lentivirus, while the cells in the shmiR-21a-5p group received Lv-mmu-miR-21a-5p knockdown, Lv-miR-21a-5p group received Lv-mmu-miR-21a-5p-overexpression.For 12–16 h, and the cells were cultured in an improved infection solution. A regular medium containing serum was then utilized to replace the cell culture medium. After 24 h, the cells were washed twice in PBS, and the 48-h puromycin (3 μg/ml) was used for screening.

The rate of green fluorescent protein-positive cells was then calculated using a fluorescence microscope for assessing transfection efficiency. To confirm the knockdown of miR-21a-5p, reverse transcription quantitative polymerase chain reaction (qRT-PCR) was employed for detecting the target genes STAT3, PTEN, and SMAD7. To confirm the overexpression of miR-21a-5p, reverse transcription quantitative polymerase chain reaction (qRT-PCR) was employed for detecting miR-21a-5p.

##### Effects of M1-Exos Derived miR-21a-5p on BMSCs Osteogenic Differentiation

In four groups of cells, 1 × 10^6^ RAW264.7 cells were seeded per well in 6-well plates. The cells were induced into M1 respectively. The exosomes were generated and named shNC, shmiR-21a-5p, Lv-NC, and Lv-miR-21a-5p. QRT-PCR was used to confirm the expression of miR-21a-5p in exosomes (Horwood, 2016)**.**

The exosomes were added to the osteogenic medium used to culture BMSCs at a concentration of 1 μg/ml 7 days later, the cells were harvested in order to be stained for alkaline phosphatase (ALP) and qRT-PCR analysis. The cells were again harvested after 14 days to be stained with alizarin red S and activity analysis.

### Flow Cytometric Analysis

Flow cytometry was used to examine the cell surface markers of BMSCs and RAW 264.7 cells. RAW264.7 macrophage cells were inoculated into a six-well plate with 1 × 10^6^ cells inoculated per well. The cells incubated with 20 ng/ml IFN-γ and 100 ng/ml LPS were named as group M1, and group M2 was incubated with 20 ng/ml IL-4, group M0 was cultured with complete medium.

After 24 h, RAW 264.7 cells were digested by trypsin and the supernatant was removed after centrifugation. The three groups (M0, M1, M2) were then suspended with 1 ml PBS and added to 5 μl APC anti-mouse CD206 and FITC anti-mouse CD86 (Biolegend, San Diego, CA, United States). APC anti-mouse CD206 was employed for intracellular flow cytometry. The cells were resuspended with 0.5 ml permeabilization wash buffer (1 x) and centrifuged at 350 g for 5 min, discarding the supernatant later. Resuspension of the cells was done with 1 ml PBS and 5 μl APC anti-mouse CD206 was added.

The antibodies utilized for identifying BMSCs are mentioned as follows: PE/Cy5 anti-mouse CD44 Antibody, APC anti-mouse CD106 Antibody, FITC anti-mouse CD45 Antibody (BioLegend, San Diego, CA, United States). These antibodies were then suspended together with 1 ml PBS.

Cell antigen staining was analyzed using a FACSC alibur analyzer and Cell Quest software (BD Biosciences).

### Quantitative Real-Time Polymerase Chain Reaction

For cellular mRNA, the RNA pure complete RNA quick isolation kit (BioTeke, Beijing, China) was used, whereas Trizol (Invitrogen, United States) was employed for miRNA, thereby completing the extraction of total RNA. First-strand cDNA was synthesized using PrimeScript™ RT Master Mix Kit (TaKaRa Bio Inc., Shiga, Japan) and the first-strand cDNA of miRNA Synthesis (stem ring method) (Sangon Biotech Co., Ltd. Shanghai, China). Later, using an Applied Biosystems 7500HT Real-Time PCR machine and SYBR PremixExTaqTMII, qRT-PCR analysis was carried out (Tli RNaseH Plus; TaKaRa, Tokyo, Japan). Primer sequences used in this study are summarized in Table 1. Gene expression was normalized to GAPDH (for cellular mRNA) and U6 (for miRNA) using the ΔΔCt method.

**TABLE 1:** Primer sequences used in this study.

Gene	Primer	Sequences (5′-3′)
CD206	Forward	AGA​CGA​AAT​CCC​TGC​TAC​TG
	Reverse	CAC​CCA​TTC​GAA​GGC​ATT​C
CD86	Forward	CTG​CTC​ATC​ATT​GTA​TGT​CAC
	Reverse	ACT​GCC​TTC​ACT​CTG​CAT​TTG
Runx2	Forward	AAA​TGC​CTC​CGC​TGT​TAT​GAA
	Reverse	GCTCCGGCCCACAAATCT
OCN	Forward	CCG​GGA​GCA​GTG​TGA​GCT​TA
	Reverse	AGG​CGG​TCT​TCA​AGC​CAT​ACT
BMP-2	Forward	TGA​CTG​GAT​CGT​GGC​ACC​TC
	Reverse	CAG​AGT​CTG​CAC​TAT​GGC​ATG​GTT​A
ALP	Forward	AGG​GTG​GGT​AGT​CAT​TTG​CAT​AG
	Reverse	GAG​GCA​TAC​GCC​ATC​ACA​TG
OPN	Forward	ATC​TCA​CCA​TTC​GGA​TGA​GTC​T
	Reverse	TGT​AGG​GAC​GAT​TGG​AGT​GAA​A
STAT3	Forward	ATTAA GGGCA GTGAG GACAT
	Reverse	GCCTT GCCTT CCTAA ATACC
PTEN	Forward	AATTC CCAGT CAGAG GCGCT ATGT
	Reverse	GATTG CAAGT TCCGC CACTG A
SMAD7	Forward	GCTAT TCCAG AAGAT GCTGT TC
	Reverse	GTTGC TGAGC TGTTC TGATT TG
miR-21a-5p	Forward	CGCTAG CTTATCAGAC TGA
	Reverse	CTCAACTGGTGTCGTGGA
miR-146a-5p	Forward	CGCTGAGA ACTGAATTCC A
	Reverse	CTCAACTGGTGTCGTGGA
miR-3473b	Forward	CGAGGGCT GGAGAGATG
	Reverse	CTCAACTGGTGTCGTGGA
U6	Forward	CTCGCTTCGGCAGCACA
	Reverse	AAC​GCT​TCA​CGA​ATT​TGC​GT
GAPDH	Forward	TGA​CCA​CAG​TCC​ATG​CCA​TC
	Reverse	GAC​GGA​CAC​ATT​GGG​GGT​AG

### Fluorescence Staining and Imaging

Macrophage polarization (M0, M1, and M2) was identified by fluorescence staining and imaging. The three groups of cells were fixed for 30 min at room temperature in 4% paraformaldehyde (MP Biomedicals, Santa Ana, CA, United States). 0.1% Triton X-100 (Sigma-Aldrich, St. Louis, MO, United States) in PBS containing 0.1% Tween-20 was used to permeabilize the cells, after washing them with PBS thrice (5 min each). The cells were blocked with 3% BSA (Sigma-Aldrich) for 30 min at room temperature. After that, samples were allowed to incubate overnight at 4°C with primary antibodies, which included rabbit anti-iNOS IgG (Abcam, United States) and mouse anti-Arginase (Abcam, United States), Anti-CD86 Antibody (Abcam, United States), and Anti-Mannose Receptor Antibody (Abcam, United States) (Abcam, United States). Secondary antibodies included Cy3-labeled Goat Anti-Rat IgG (Beyotime, Shanghai, China), Cy3-labeled Goat Anti-Mouse IgG (Beyotime, Shanghai, China), and Alexa Fluor 488-labeled Goat Anti-Mouse IgG (Beyotime, Shanghai, China), and cells were counterstained with 6-diamidino-2-phenylindole (DAPI) (Beyotime, Shanghai, China) solution at 1 μg/ml. Images were obtained using a fluorescence microscope was used to capture the images (BX51; Olympus, Tokyo, and Japan).

### Statistical Analyses

All data are presented as the mean and standard deviation (±SD) from at least three independent experiments. Statistically signifcant differences were determined by one-way ANOVA and independent unpaired parametric 2-tailed Student’s *t* test. All statistical analysis was performed on GraphPad Prism 7 (GraphPad Software). *p* values of less than 0.05 were considered significant and denoted with an asterisk.

## Results

### Characterization of Polarized Macrophages

Flow cytometry, qRT-PCR, and immunofluorescent staining were used to assess the expression of phenotype markers associated with M1 and M2 macrophages. RAW264.7 induced by IFN-γ plus LPS was named as group M1, and RAW264.7 induced by IL-4 was named as group M2. Cells were incubated in a normal complete medium as an unpolarized phenotype (M0). CD86 and CD206 being M1 and M2 surface markers, respectively. Flow cytometric analysis revealed a stronger CD86 expression in M1 whereas CD206 was highly expressed in M2 (Additional file: Supplementary Figure S1A). Analysis of mRNA expression of CD86 and CD206 in macrophages was carried out using qRT-PCR. Our results demonstrated a significant rise in the CD86 mRNA expression in M1 macrophages. In addition, CD206 was found to be significantly up-regulated in M2 macrophages (Additional file: Supplementary Figure S1B). An immunofluorescence assay was used to confirm the polarization of macrophages. iNOS and CD86 (M1-specific markers) staining was observed to be much stronger in M1 macrophages whereas the staining of Arg-1 and CD206 (M2-specific markers) was manifested much strongly in M2 macrophages (Additional file: Supplementary Figure S1C). We successfully induced M1 and M2 phenotypes in macrophages.

### Identification of Exosomes Generated by Macrophages

**FIGURE 1:**
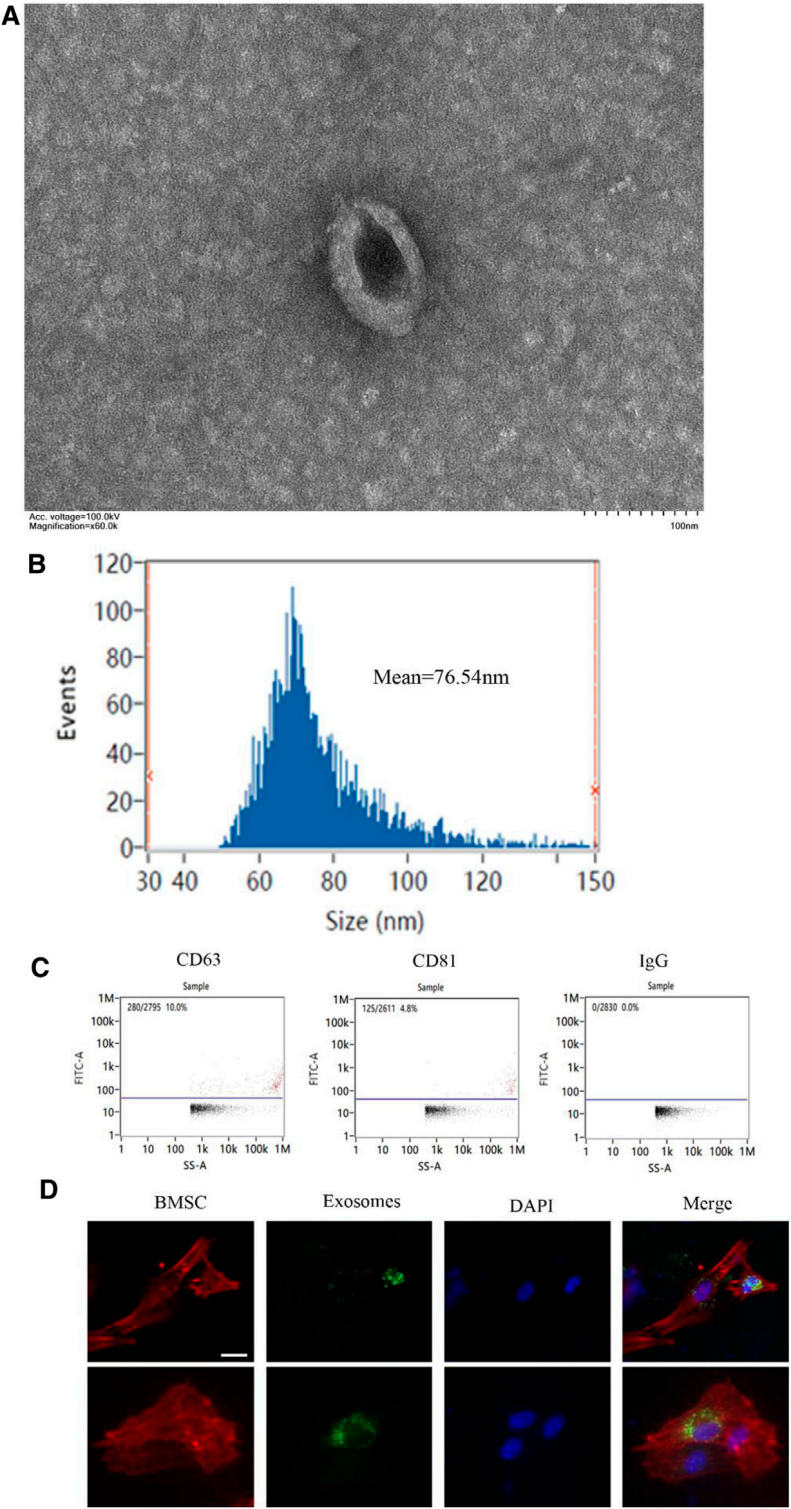
Identification of exosomes generated by macrophages. **(A)** The morphology of exosomes under transmission electron microscope (scale bar: 100 nm); **(B)** The size distribution curve of the exosomes was determined by nanoparticle tracking analysis; **(C)** The expression of exosome marker proteins CD81 and CD63 was analyzed by flow cytometry; **(D)** Representative immunofluorescence images showing the internalization of PKH67-labeled BMDM-derived exosomes (green) by BMSCs stained with phalloidine (red) at 6 h. Cell nuclei were stained with DAPI (blue), white arrows indicate exosomes (green). Scale bars, 10 μm. *p <0.05 and **p < 0.01.

The exosomes were extracted from M1 and M2 macrophages’ CM. Transmission electron microscopy was employed to examine the morphology of exosomes. As shown in Figure 1A, exosomes were elliptical vesicles with bilayer membranes (Figure 1A). These exosomes have a diameter of 30–150 nm, according to nanoparticle tracking analysis (Figure 1B). Exosomal markers CD81 and CD63 were clearly expressed by flow cytometry for nanoparticle analysis (Figure 1C). Fluorescence microscope imaging revealed the presence of PKH67 spots in recipient fibroblasts after incubation, indicating that macrophage's exosomes were delivered to BMSCs (Figure 1D).

### M1-Exos Promote Osteogenic Differentiation of BMSCs

**FIGURE 2:**
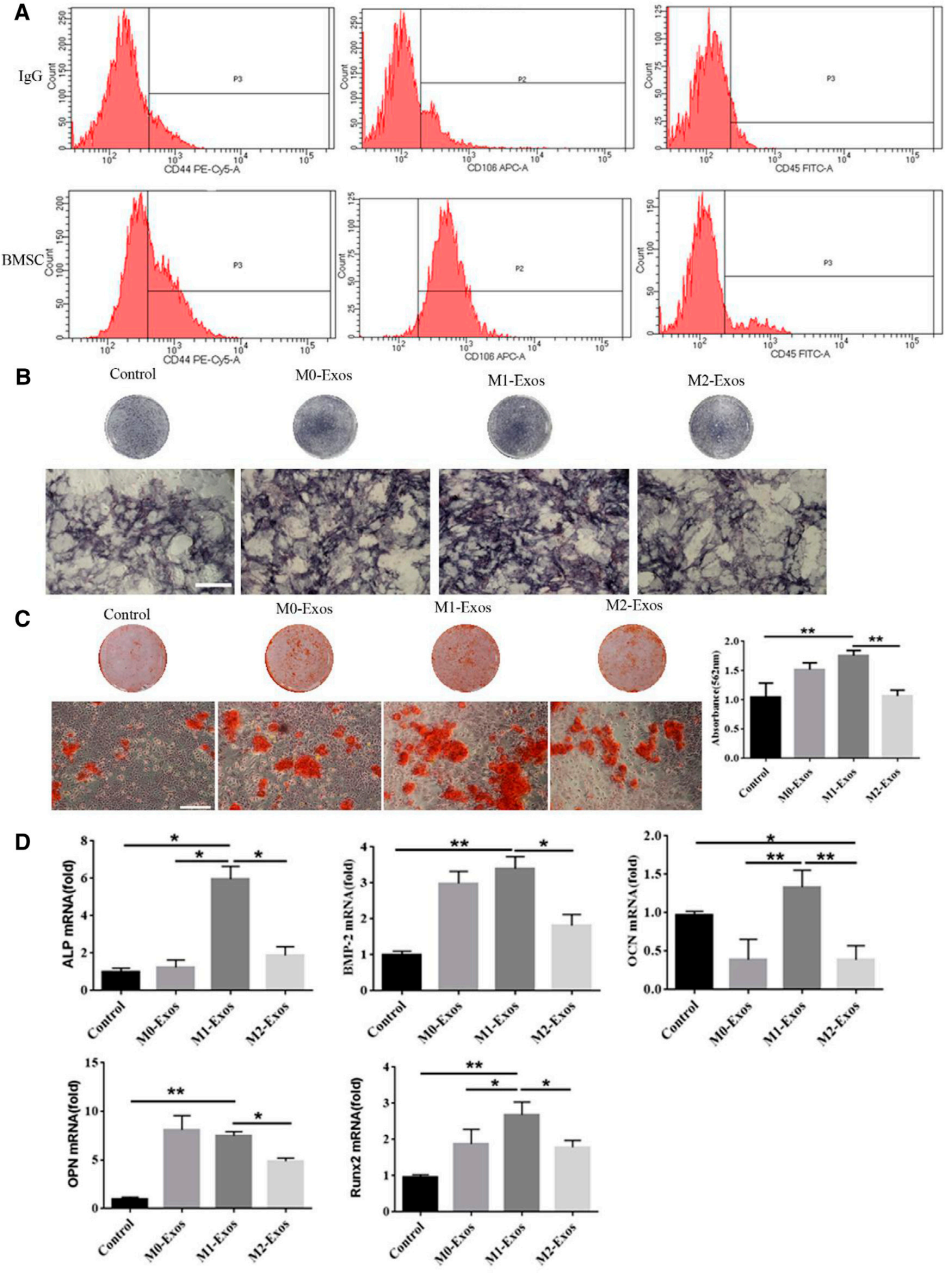
M1-Exos promote osteogenic differentiation of BMSCs. **(A)** Identification of BMSCs markers by flow cytometry. **(B)** ALP stainingand in BMSCs (Scale bars, 100 μm). **(C)** Alizarin red S staining and quantification in BMSCs (Scale bars, 100 μm); **(D)** The expression of osteogenic gene was detected by qRT-PCR. Data are presented as mean ± SD. *p < 0.05, **p < 0.01 represent significant differences between the indicated columns.

Next, we extracted mouse BMSCs and the expression of markers was identified by flow cytometry studied. The results indicated that BMSCs were successfully extracted treated (Figure 2A). The impact of exosomes secreted by macrophages on osteogenic differentiation of BMSCs was investigated. For the incubation of BMSCs, M0-Exos, M1-Exos, and or M2-Exos were added to the osteogenic medium. The control group received the same amount of osteogenic medium. In BMSCs treated with Exos-based osteogenic medium for 7–14 days, we measured ALP activity, osteogenesis-related gene expression, and alizarin red S staining. The results indicated that M1 and M2 polarized macrophage exosomes all can promote osteogenesis of BMSCs. In M1-Exos, there were more ALP-positive cells (Figure 2B). M1-Exos BMSCs formed more calcium deposits than other types, according to Alizarin Red S staining. According to quantitative analysis, BMSCs in M1-Exos provided the most mineralized nodules (Figure 2C). Expression of the osteogenic genes ALP, BMP-2, OPN, OCN, and Runx2 were then measured by qRTPCR. Compared with other groups, the mRNA levels of these genes were significantly increased in M1-Exos group (Figure 2D). This finding further indicated that M1 may promote the early stages of osteogenesis mainly through exosomes.

### Differential miRNAs of M1-Exos and M2-Exos were Detected by Small RNA-seq Technology

**FIGURE 3:**
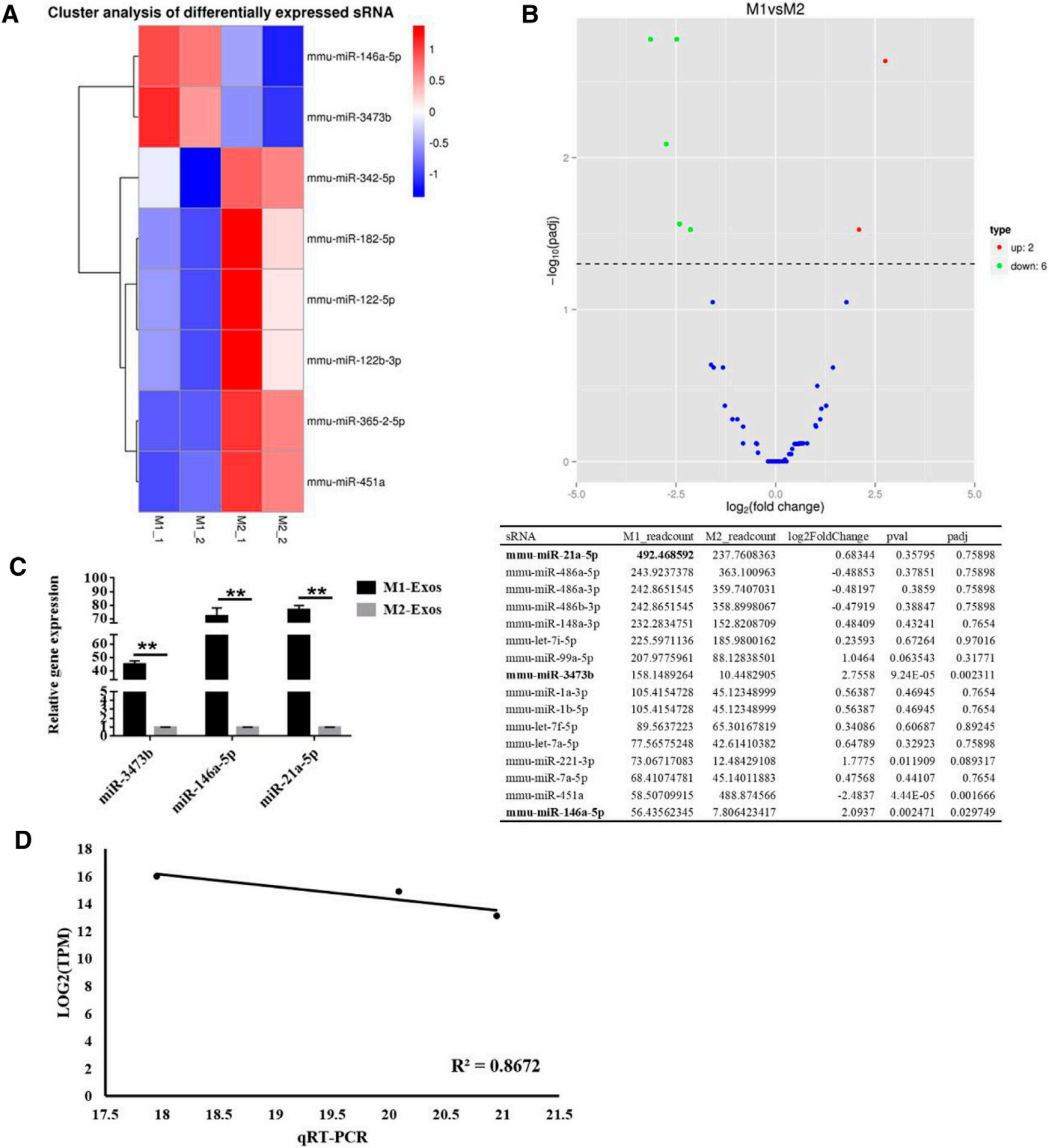
Identifcation of diferentially expressed miRNAs between M1-Exos and M2-Exos. **(A)** Heat map identifed the diferently expressed miRNAs of M1-Exos and M2-Exos. Red and blue were used to indicate the up-regulated and down-regulated genes respectively. *n* = 2 for each group. M1, Exosomes were extracted from medium of M1 macrophage medium; M2, Exosomes were extracted from medium of M1 macrophage medium; **(B)** Volcano plot comparing the levels of gene expression between M1-Exos and M2-Exos. Red and green dots represent upregulated and downregulated miRNAs (>2.0 fold change and *p* < 0.05). **(C)** The expression of miR-3473b, miR-146a-5p and miR-21a-5p was detected by qRT-PCR. Data are presented as mean ± SD. **p < 0.01 represent significant differences between the indicated columns. **(D)** Gene expression correlation between qRT-PCR and RNA-seq data. Te Pearson correlation coefficients and linear regression line are indicated.

To explore the mechanism of M1-Exos promoting osteogenesis of BMSCs, we made use of miRNA microarray for comparing the differentially-expressed miRNAs in M1-Exos and M2-Exos. The difference of miRNA expression between M1-Exos and M2-Exos was detected by independent hierarchical clustering and demonstrated using a heat map (Figure 3A). As demonstrated in Figure 3B, a volcano plot illustrates the expression variance in the number of differentially-expressed miRNAs at different *p*-values and fold changes. Based on a threshold set at (>2.0 fold change and *p* < 0.05) for the microarray data, a total of 8 differentially expressed mRNAs were identified in M1-Exos in comparison with the M2-Exos macrophage samples, of which two miRNAs (miR-3473b, miR-146a-5p) were up-regulated whereas six of them (miR-342-5p, mmu-miR-451a, miR-365-2-5p, miR-182-5p, miR-122-5p, and miR-122b-3p) were down-regulated. Although microarray analysis showed that miR-21a-5p had no significant difference M1-Exos and M2-Exos, it was the most abundant microRNA in M1-Exos (Figure 3C). Therefore, we verified the expression of miR-3473b and miR-146a-5p as well as miR-21a-5p via qRT-PCR. The results revealed that miR-21a-5p was significantly up-regulated in M1-Exos compared with M2-Exos (Figure 3C). To evaluate concordance in gene expression intensities between RNA-seq and qRT-PCR, we calculated expression correlation between qRT-qPCR C_T_-values and log transformed RNA-seq expression values (Everaert et al., 2017). High expression correlations were observed between RNA-seq and qRT-PCR expression intensities (Pearson correlation, *R* ^2^ = 0.845) (Figure 3D).

### MiR-21a-5p Induces Osteogenic Differentiation of BMSCs *In Vitro*

**FIGURE 4:**
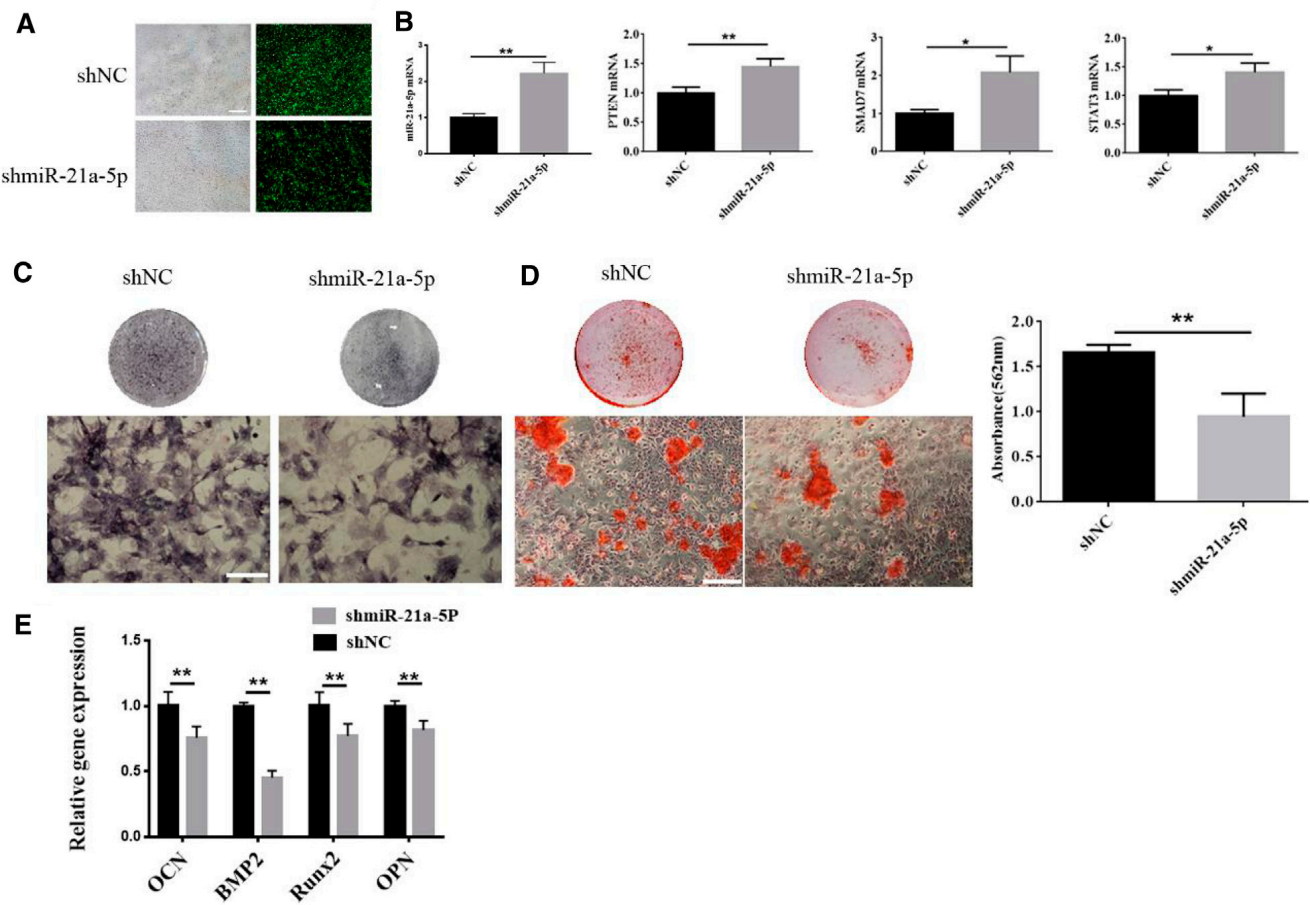
Effect of knocking down miR-21a-5p in M1 macrophages on osteogenic differentiation of BMSCs. **(A)** sh-miR-21a-5p were knocked down using a lentiviral transfection technique. **(B)** qPT-PCR was used to verify the expression of miR-21a-5p, SMAD7, PTEN, and STAT3. **p < 0.01. **(C)** ALP staining in BMSCs (Scale bars, 100 μm). **(D)** Alizarin red S staining and quantification in BMSCs (Scale bars, 100 μm). **(E)** The expression of osteogenic gene was detected by qRT-PCR (7 days).Data are presented as mean ± SD. *p < 0.05, **p < 0.01 represent significant differences between the indicated columns.

**FIGURE 5:**
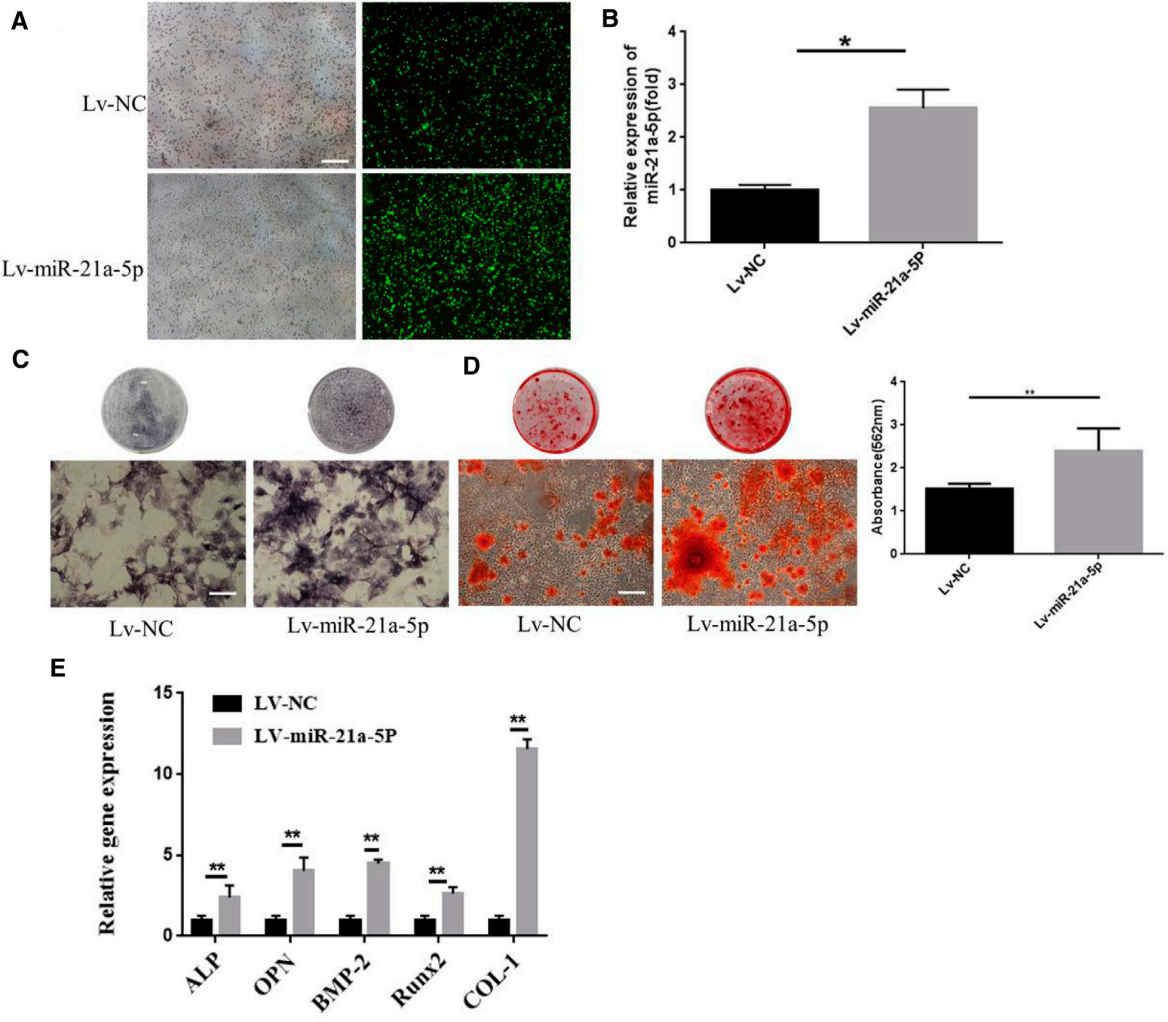
Effect of miR-21a-5p overexpression in M1 macrophages on osteogenic differentiation of BMSCs. **(A)** MiR-21a-5p was overexpressed using lentivirus transfection technique. **(B)** QPT-PCR was used to verify the expression of miR-21a-5p. **(C)** ALP staining in BMSCs (Scale bars, 100 μm). **(D)** Alizarin red S staining and quantification in BMSCs (Scale bars, 100 μm). **(E)** The expression of osteogenic gene was detected by qRT-PCR. Data are presented as mean ± SD. *p < 0.05, **p < 0.01 represent significant differences between the indicated columns.

We intended to investigate whether miR-21a-5p was a crucial genetic factor that influenced BMSCs osteogenic differentiation since it was the most abundant microRNA and its levels were found to increase dramatically in M1-Exos (Zhang et al., 2017). Lentivirus transfection was utilized for knocking down miR-21a-5p in M1 macrophage and the knockdown rate was 80%, thereby suggesting that individual genes have been successfully knocked down (Figure 4A). qRT-PCR verified the expression of miR-21a-5p′s target genes SMAD7, PTEN, and STAT3. The outcome revealed that the gene expression of SMAD7, PTEN, and STAT3 increased after the knockdown of mir-21a-5p (Figure 4B). M1 transfected with shmiR-21a-5p, as well as M1 with shNC were cultured in a 10 cm culture dish. The exosomes were extracted from the supernatant of each group and added to the osteogenic induction medium to incubate BMSCs. The results demonstrated that there were fewer ALP positive cells in the shmiR-21a-5p group (Figure 4C). Alizarin red S staining revealed that the shmiR-21a-5p group had the least calcium deposition of all, and the quantitative analysis showed that the mineralized nodules were also the least (Figure 4D). The expression of osteogenic genes OCN, BMP-2, ALP, OPN, and Runx2 was detected by qRT-PCR. In comparison to shNC, the mRNA levels of these genes in the shmiR-21a-5p group were substantially lower (Figure 4E). Next, we used the same method to overexpress miR-21a-5p to detect osteogenic differentiation of BMSCs (Figure 5A). The outcome revealed that the gene expression of miR-21a-5p increased (Figure 5B). Moreover, there were more ALP positive cells in the Lv-miR-21a-5p group (Figure 5C). Alizarin red S staining showed the most calcium deposition in the Lv-miR-21a-5p group, and quantitative analysis showed the most mineralized nodules (Figure 5D). The expression of osteogenic genes ALP, OPN, BMP2, Runx2, and COL-1 was detected by qRT-PCR (7 days). The mRNA levels of these genes were significantly higher in the Lv-miR-21a-5p group compared to Lv-NC (Figure 5E).

## Discussion

Bone defect repair has always been a difficult problem in clinical treatment, due to the destruction of the inflammatory microenvironment. Macrophages have an indispensable role in the control of bone regeneration in normal bones, contributing to inflammation and debridement of the injury site (Pajarinen et al., 2019). Macrophages can polarize from a nonpolarized state to M1 and M2, making them important targets for therapeutic. Studies have shown that macrophage polarization can promote osteoblast differentiation (Horwood, 2016). MSCs co-cultured with M2 macrophages results in substantially increased MSCs mineralization due to soluble factors, according to studies. M0 and M1 macrophages, in particular, only stimulated MSCs osteogenic differentiation in the early stage of co-culture (Zhang et al., 2017). The significance of an original, transient inflammatory process mediated by M1 macrophage-BMSCs cross-talk in enhancing osteoblast differentiation was highlighted by our findings. Lu et al. found that exposing BMSCs to a high density of pro-inflammatory M1 macrophages stimulated them to achieve their maximum pro-osteogenic capacity and immune-modulatory effect by reciprocally modulating an ideal transfer of macrophages from M1 to M2 phenotype for optimal bone healing (Lu et al., 2017). Zhang et al. co-cultured MSCs with M0, M1, and M2 macrophages and measured ALP activity at 7, 14, and 28 days, respectively. The results revealed that M1 macrophages and M2 macrophages had the advantage of promoting osteogenic differentiation at 7 and 28 days, respectively, (Zhang et al., 2017).

Macrophage-derived exosomes have become a research hotspot in recent years. Exosomes, which are secreted by almost all cells and widely present in various body fluids, are nano-sized vesicles of 30–150 nm in diameter. Exosomes from various kinds of cells contain 194 lipids, 4,400 proteins, 764 miRNAs, and 1,639 mRNAs approximately, suggesting their complexity as well as the diversity of function (Pegtel and Gould, 2019; Zhang et al., 2019). The influence of M1 and M2 exosomes upon BMSCs osteogenic differentiation were investigated. The exosomes secreted by M0, M1, M2 were added to the osteogenic induction medium of BMSCs for 7 and 14 days and macrophage exosomes were all found to facilitate osteogenic differentiation of BMSCs. And M1 exosomes are more effective. This is consistent with the research results of Xia et al. (2020). Upon bone injury, monocytes are recruited to the wound site and differentiate into activated macrophages. In the early stages of injury mainly polarized to M1 macrophages (Vallés et al., 2020). We speculate that the M1 phenotype is essential for early osteogenic differentiation. In exosomes, 80% of the content is miRNA. Exosomes, which are secreted by macrophages, enhance the inflammatory response of receptor cells by controlling the levels of microRNAs (miRNAs) and play an important role in cell-to-cell contact (Li et al., 2019). We used microarray analysis to discover that miR-21a-5p is the main miRNA enriched in M1 exosomes, which may play a role in bone formation/regeneration promotion or inhibition. We knocked down and overexpressed miR-21a-5p in M1 macrophages to verify whether miR-21a-5p affects the osteogenic differentiation of BMSCs. The results showed that when BMSCs were incubated with exosomes secreted by M1 macrophages with a miR-21a-5p knockdown, the number of ALP positive cells and mineralized nodules were decreased, and the expression of osteogenic related genes was decreased. Meanwile, M1 macrophage exosomes overexpressing miR-21a-5p promoted the osteogenic differentiation of BMSCs. The present data indicates that M1 macrophagy-derived exosomes miR-21a-5p induces BMSCs towards osteoblastic fate in the early stage of osteogenesis. The findings suggested that the M1 macrophage-BMSCs crosstalk could aid osteogenesis by stimulating osteoblast differentiation through paracrine signaling.

Many studies have demonstrated the effects of MSCs priming with pro-inflammatory cytokines or growth factors (Noronha et al., 2019). This is consistent with our point of view. In addition, exosomes were added at a low concentration of 1 μg/ml. One study indicated that low-inflammatory macrophages could activate autophagy in BMSCs to improve osteogenesis (Yang et al., 2021).These observations highlight the importance of an initial, transient inflammatory phase during bone healing.

Modern research showed that exosome is an effective and potent additive to produce advanced immunomodulatory and bone regeneration materials. There are results showed the exosomes loaded sulfonated polyetheretherketone (SPEEK) promoted macrophage polarization via the NF-κB pathway to enhance BMSCs osteogenic differentiation (Fan et al., 2021). So, the application of bone biomaterials combined with exosomes in bone tissue engineering may be promising.

The findings that co-culturing of BMSCs with exosomes secreted by M1 macrophages enhances osteogenesis of the BMSCs suggest the feasibility of targeting BMSCs-macrophage crosstalk for bone repair. Besides, the mechanism of M1 macrophage-derived exosomes miR-21a-5p inducing osteogenic differentiation of BMSCs needs further study and should be modeled by animal experiments, so as to open up a new therapeutic scheme for macrophages to repair bone defects.

## Conclusion

The effect of macrophage exosomes on BMSCs osteogenic differentiation was investigated in this research. The results indicated that M1 and M2 polarized macrophage exosomes all can promote osteogenesis of BMSCs. Especially, M1 macrophage exosomes have a stronger ability at the early stage of inflammation.The enhanced osteogenesis mediated by M1 macrophage exosomes is related to the miR-21a-5p. Understanding the regulatory effect of M1 macrophage exosome miRNA on BMSCs will aid in designing effective strategies to guide the fate of BMSCs and improve the regeneration results and have immense potential for optimizing fracture therapies.

## Data Availability

The original contributions presented in the study are included in the article/Supplementary Material, further inquiries can be directed to the corresponding authors.
