# Supplementary material for: Macrophage-Derived Exosomes Promote Bone Mesenchymal Stem Cells Towards Osteoblastic Fate Through microRNA-21a-5p
Source: Front Bioeng Biotechnol. 2022 Jan 5;9:801432. doi: 10.3389/fbioe.2021.801432 (PMC8766741; doi:10.3389/fbioe.2021.801432)
Supplement: Supplementary file 1 [file DataSheet1.pdf]

A

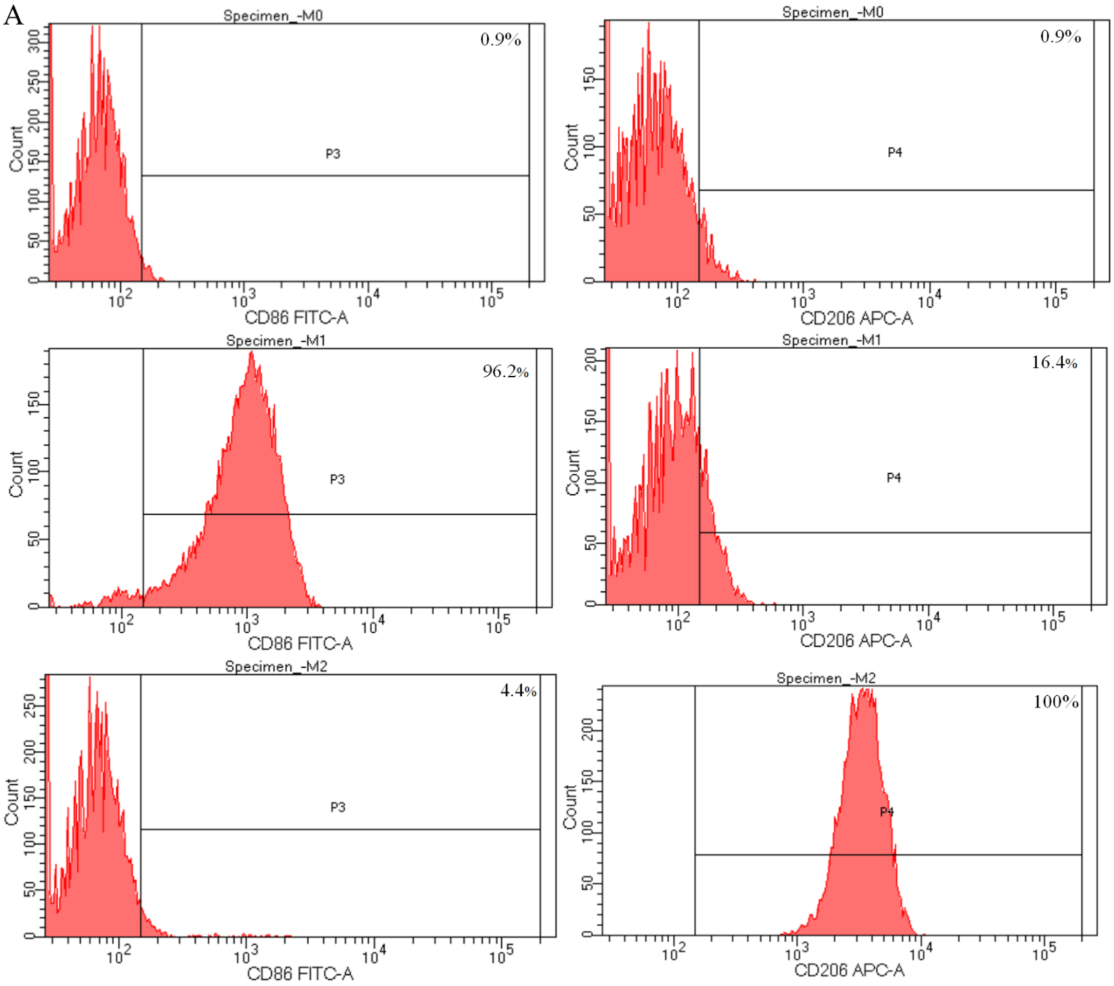

Specimen_-M0
0.9%
P3
Count
CD86 FITC-A
Specimen_-M0
0.9%
P4
Count
CD206 APC-A
Specimen_-M1
96.2%
P3
Count
CD86 FITC-A
Specimen_-M1
16.4%
P4
Count
CD206 APC-A
Specimen_-M2
4.4%
P3
Count
CD86 FITC-A
Specimen_-M2
100%
P4
Count
CD206 APC-A

B

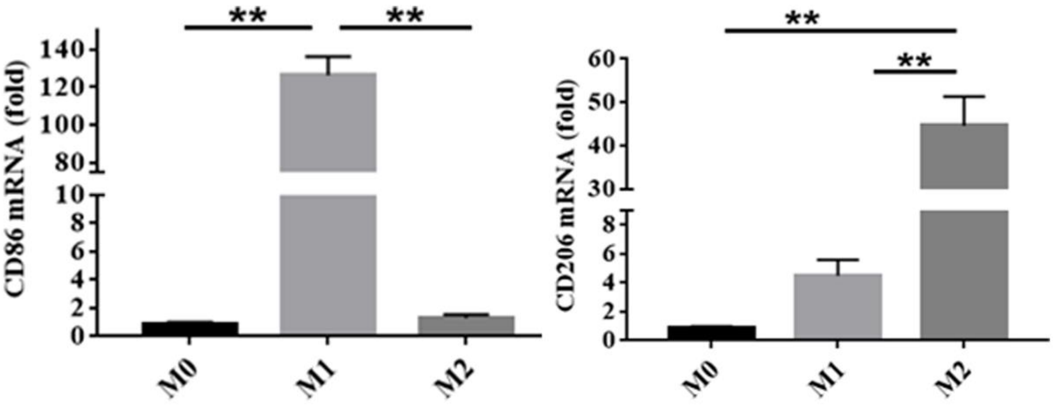

CD86 mRNA (fold)
**
**
M0
M1
M2
CD206 mRNA (fold)
**
**
M0
M1
M2

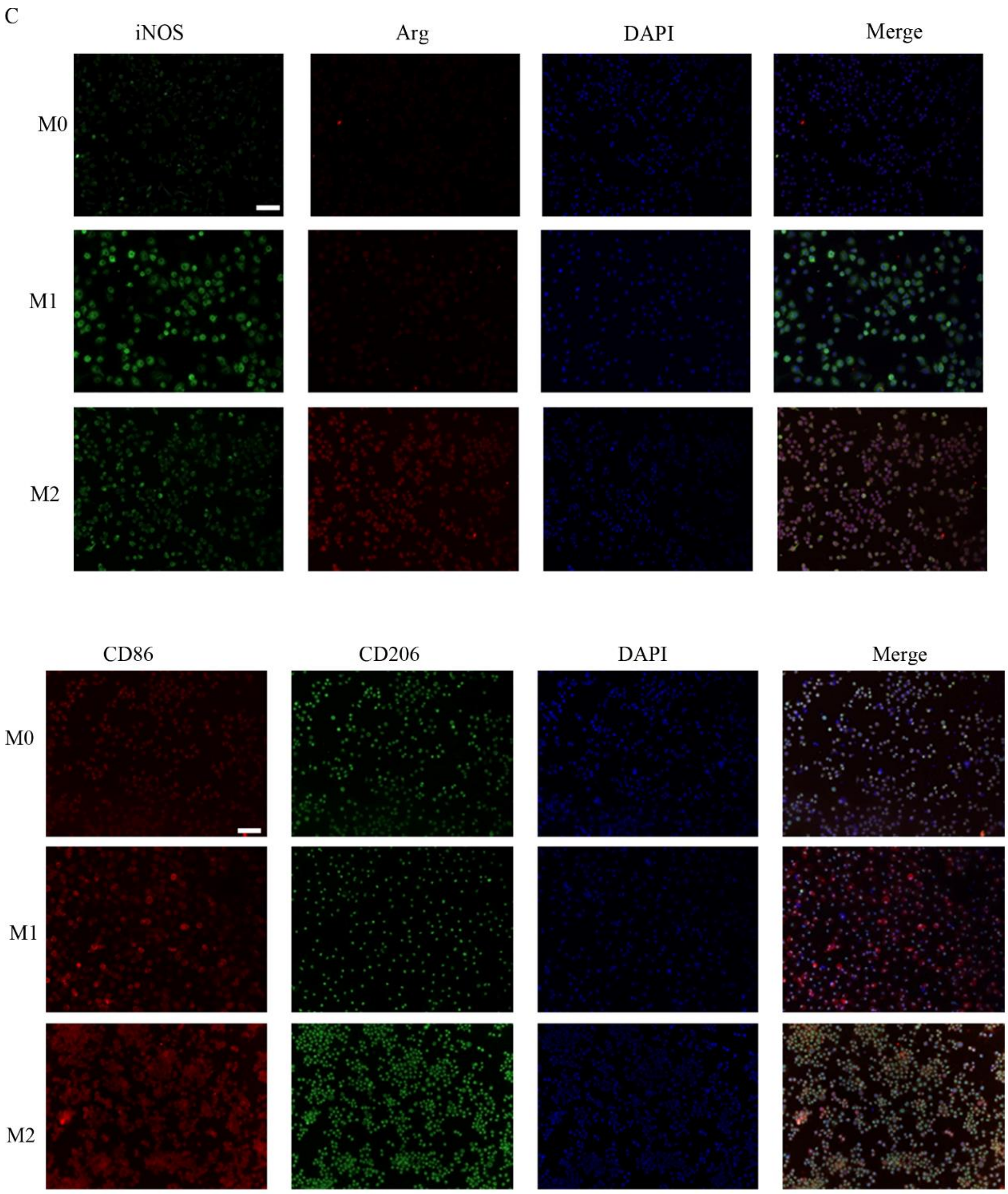

Fig S1. Characterization of M1 and M2 Macrophages. (A) Te expression of CD86 and CD206 on macrophages was analyzed by flow cytometry analysis. (B) M1 and M2 makers were assessed by qRT-PCR. Data are presented as the mean±SD; **$P<0.01$ represent significant differences between the indicated columns. (C) Immunofluorescent staining of M1 and M2 markers in polarized macrophages (scale bar: 50 μm).
